# Adaptive Microwave Staring Correlated Imaging for Targets Appearing in Discrete Clusters

**DOI:** 10.3390/s17102409

**Published:** 2017-10-21

**Authors:** Chao Tian, Zheng Jiang, Weidong Chen, Dongjin Wang

**Affiliations:** Key Laboratory of Electromagnetic Space Information, Chinese Academy of Sciences, University of Science and Technology of China, Hefei 230027, China; jiangz10@mail.ustc.edu.cn (Z.J.); wdchen@ustc.edu.cn (W.C.); wangdj@ustc.edu.cn (D.W.)

**Keywords:** microwave staring correlated imaging, adaptive imaging, discrete cluster, target strip, spectral entropy, band exclusion

## Abstract

Microwave staring correlated imaging (MSCI) can achieve ultra-high resolution in real aperture staring radar imaging using the correlated imaging process (CIP) under all-weather and all-day circumstances. The CIP must combine the received echo signal with the temporal-spatial stochastic radiation field. However, a precondition of the CIP is that the continuous imaging region must be discretized to a fine grid, and the measurement matrix should be accurately computed, which makes the imaging process highly complex when the MSCI system observes a wide area. This paper proposes an adaptive imaging approach for the targets in discrete clusters to reduce the complexity of the CIP. The approach is divided into two main stages. First, as discrete clustered targets are distributed in different range strips in the imaging region, the transmitters of the MSCI emit narrow-pulse waveforms to separate the echoes of the targets in different strips in the time domain; using spectral entropy, a modified method robust against noise is put forward to detect the echoes of the discrete clustered targets, based on which the strips with targets can be adaptively located. Second, in a strip with targets, the matched filter reconstruction algorithm is used to locate the regions with targets, and only the regions of interest are discretized to a fine grid; sparse recovery is used, and the band exclusion is used to maintain the non-correlation of the dictionary. Simulation results are presented to demonstrate that the proposed approach can accurately and adaptively locate the regions with targets and obtain high-quality reconstructed images.

## 1. Introduction

Microwave staring correlated imaging (MSCI), also called radar coincidence imaging (RCI), was recently proposed as a new microwave imaging method [1,2,3,4]. The essence of MSCI is to construct temporal-spatial stochastic radiation field in the imaging region, which is typically realized by a multi-transmitter configuration emitting independent stochastic waveforms. The stochastic waveforms can be transimitted directly from noise-generating microwave sources, or generated by the transmitters using random phase and frequency modulation [2,5,6]. In comparison with the conventional multiple-input multiple-output (MIMO) radar, which transmits orthogonal waveforms with each transmitter, MSCI focuses on stochastic radiation fields in the imaging plane. Achieving channel separation via waveform orthogonality is not required throughout the MSCI procedure, unlike MIMO radar [3]. With random radiation from multiphase centers, MSCI can increase the resolution of the targets within the same beam coverage under all-weather and all-day circumstances using the correlated imaging process (CIP), which requires the received signal to be combined with the temporal-spatial stochastic radiation field.

MSCI has attracted increasing attention in related research fields. In [7], MSCI was extended to 3D imaging and proven to be able to provide target 3D images using a single pulse without the requirement for motion parameter knowledge. Because there is generally a gain-phase error, which is difficult to have accurate knowledge of, a sparse auto-calibration method was proposed in [8] to perform gain-phase error calibration in the sparsity-driven RCI. To improve the independence degree of the radiation field, Bo Liu et al. presented a new type of ideal independent radiation field: the orthogonal radiation field for MSCI [9]. In [10], a novel adaptive clustered sparse Bayesian learning algorithm was proposed for extended target imaging. The algorithm exploits the continuity structure of the extended target. In [3], a typical MSCI system was designed, and an outfield experiment was performed to verify the effectiveness of the MSCI scheme.

Before the CIP, the MSCI must discretize the continuous target region to a fine grid and accurately compute the measurement matrix [1,2,3,4,7,8,9,10]. The MSCI system typically observes a wide area [1,2,11]; thus, using a uniform fine grid in the entire imaging region will generate an excessive number of grids, which makes the imaging process highly complex and in turn limits the real-time imaging performance. When the targets appear in discrete clusters, such as boats in a calm bay, vehicles in an open-air parking lot, the majority of the observation region does not exhibit scatterers, and, thus, grid subdivision will not benefit the imaging process. To reduce the complexity of the imaging process, the correlation method was employed to first estimate the subareas where the targets exist [11]. On this basis, this paper focuses on adaptive MSCI for targets in discrete clusters and uses narrow pulses of a stochastic signal to first locate target-containing strips in the range direction, which further reduces the complexity of the CIP.

The transmitters of MSCI typically emit pulse waveforms [2,3]. In this paper, we use narrower pulse waveforms to detect the imaging region. Discrete clustered targets are distributed in different range strips in the imaging region, and these strips are illuminated in different time periods when the pulse of the waveform is narrow. Hence, the system separates the echoes of the targets in different strips in the time domain. Here, a modified method that is robust against noise based on the spectral entropy [12] is put forward to detect the echoes of the discrete clustered targets; this method enables the strips with targets to be located in an adaptive manner.

In a strip with targets, the matched filter (MF) reconstruction algorithm [13] is used to locate the regions with targets, and only the regions of interest are discretized to a fine grid. Grid refinement generates more grids than the measurements, and the target scatterers are often sparsely distributed [8]; thus, sparse recovery is used. Because the scatterers are distributed in a continuous scene, the scattering centers are generally located off the grids, regardless of the fineness of the grid. Therefore, a gridding error is generated and is inversely proportional to the grid size. For fine grids, the increasing coherence of the dictionary makes it difficult to ensure the sparse reconstruction, which relies on a low correlation of the columns of the measurement matrix. The band exclusion (BE) [14] is used to solve this problem.

The remainder of this paper is outlined as follows. In Section 2, the MSCI mode is presented. Section 3 presents the adaptive imaging method in detail. In Section 4, the effectiveness of the proposed method is verified using numerical examples. Finally, Section 5 concludes the paper.

## 2. MSCI Model

**Figure 1:**
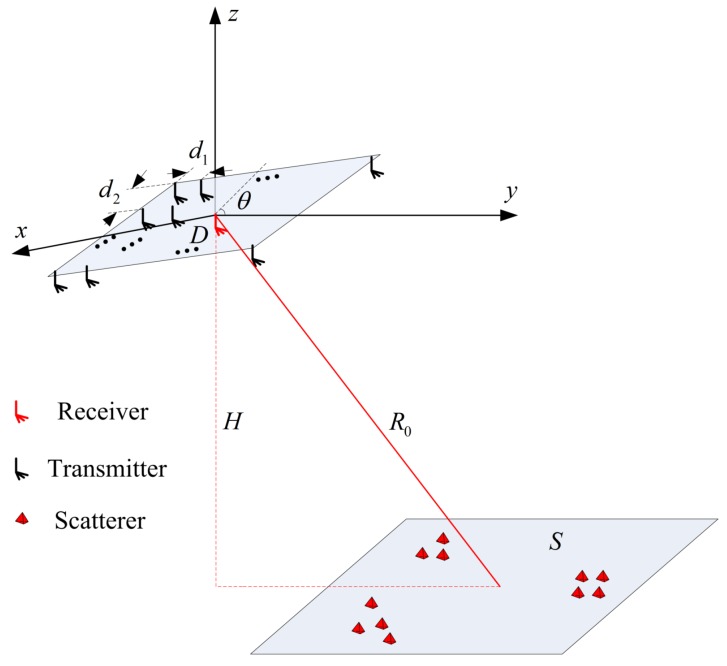
Imaging geometry of the microwave staring correlated imaging system.

MSCI can be realized using a multi-transmitter configuration to transmit time-independent and group-orthogonal waveforms [1,2,4]. As shown in Figure 1, the MSCI system is generally composed of *N* transmitters and one receiver, whose position vectors are denoted by ${\vec{r}}_{i}^{t}$ and ${\vec{r}}^{r}$ , respectively. The scatterers are distributed in the imaging region *S*; ${\vec{r}}_{l}$ represents the position vector of the *l*-th scatterer. *S* is in the *XY* plane, which is *H* below the origin. $R_{0}$ is the distance from the center of the imaging region to the origin. θ is the angle between antenna aperture *D* and the *XY* plane.

The correct model for MSCI is Maxwell’s equations, but the simpler scalar wave equation and first-order Born approximation are commonly used; thus, the echo $E^{sca}\left(t\right)$ is modeled as [1,2,15]

(1) $$E^{sca}\left(t\right)=\int_{S}\frac{1}{4\pi \left|\vec{r}-{\vec{r}}^{r}\right|}\sum_{i}\frac{St_{i}\left(t-\frac{\left|\vec{r}-{\vec{r}}_{i}^{t}\right|}{c}-\frac{\left|\vec{r}-{\vec{r}}^{r}\right|}{c}\right)}{4\pi \left|\vec{r}-{\vec{r}}_{i}^{t}\right|}\sigma \left(\vec{r}\right)d\vec{r},$$

where *c* and $St_{i}\left(t\right)$ are the speed of light, and excitation signal of the *i*-th transmitter, respectively. The target information in the imaging area is described by the scattering coefficients $\sigma \left(\vec{r}\right)$ . The modified radiation field is defined as

(2) Erad(r→,t)=14πr→−r→r∑iSti(t−r→−r→itr→−r→itcc−r→−r→rr→−r→rcc)4πr→−r→it

and $E^{sca}\left(t\right)$ is considered to be contaminated by noise n(t); thus, the received signal is

(3) $$E_{r}\left(t\right)=\int_{S}E_{rad}\left(\vec{r},t\right)\sigma (\overset{⇀}{r})d\overset{⇀}{r}+n(t)$$

Let $t_{k},k=1,\ldots ,K$ be the sample times. Set $E_{r}=\left(E_{r}\left(t_{k}\right)\right)\in C^{K}$ as the data vector. Then, the imaging region is discretized as

(4) $$I_{L}=\left[{\vec{r}}_{1},{\vec{r}}_{2},\ldots ,{\vec{r}}_{l},\ldots ,{\vec{r}}_{L}\right],\;l=1,2,\ldots ,L.$$

The scattering coefficient vector σ is expressed as $\sigma ={\left[\sigma_{1},\sigma_{2},\ldots ,\sigma_{l},\ldots ,\sigma_{L}\right]}^{T},\;l=1,2,\ldots ,L,$ where the components of σ are equal to the scattering coefficients when the grid points are the nearest grid points to positions $\left\{{\vec{r}}_{l}\right\}$ and zero otherwise. The dynamic range is introduced to describe the difference in scattering coefficients which is defined as $\sigma_{\max}/\sigma_{\min}$, where

(5) $$\sigma_{\max}=\max_{l}\left|\sigma_{l}\right|,\sigma_{\min}=\min_{l}\left|\sigma_{l}\right|.$$

Let the measurement matrix be

(6) $$E_{rad}=\left[\begin{array}{cccc} E_{rad}\left({\vec{r}}_{1},t_{1}\right) & E_{rad}\left({\vec{r}}_{2},t_{1}\right) & \ldots & E_{rad}\left({\vec{r}}_{L},t_{1}\right)\\ E_{rad}\left({\vec{r}}_{1},t_{2}\right) & E_{rad}\left({\vec{r}}_{2},t_{2}\right) & \ldots & E_{rad}\left({\vec{r}}_{L},t_{2}\right)\\ \ldots & \ldots & \ldots & \ldots \\ E_{rad}\left({\vec{r}}_{1},t_{K}\right) & E_{rad}\left({\vec{r}}_{2},t_{K}\right) & \ldots & E_{rad}\left({\vec{r}}_{L},t_{K}\right) \end{array}\right].$$

Then, the mathematic model of MSCI can be described in the form

(7) $$E_{r}=E_{rad}\sigma +d+n,$$

where $n=\left(n\left(t_{k}\right)\right)$ is the external noise and the gridding error is given by $d=E_{r}-E_{rad}\sigma -n$ . To estimate the scattering coefficients, the MSCI solves the inverse problem, which recovers σ from the measurement vector $E_{r}$ and dictionary $E_{rad}$.

## 3. Adaptive Imaging Method for Targets Appearing in Discrete Clusters

According to Equation (7), MSCI must discretize the continuous target space to a fine grid and accurately compute the measurement matrix. The MSCI system commonly observes a wide area; thus, using a uniform fine grid in the entire imaging area will generate an excessive number of grid-cells, which will increase the computational burden. This section will introduce an adaptive imaging method in detail, which significantly reduces the computational burden when the targets appear in discrete clusters.

The transmitters of MSCI typically emit pulse waveforms [2,3], and the waveform from the *i*-th transmitter is

(8) $$St_{i}\left(t\right)=\sum_{q=0}^{Q-1}rect\left(\frac{t-qT}{T_{p}}\right)\cdot st_{iq}\left(t\right),$$

where $T_{p}$, *T* and *Q* are the pulse width, pulse repetition period, and pulse number, respectively. In this paper, we use narrower pulse waveforms to detect the imaging region. When the pulse width $T_{p}$ is narrow, the modified radiation field $E_{rad}\left(\vec{r},t\right)$ is distributed only in a part of the imaging region at a given time, whose width δy is associated with the pulse width, δy≈12cTp12cTpsinθsinθ .

**Figure 2:**
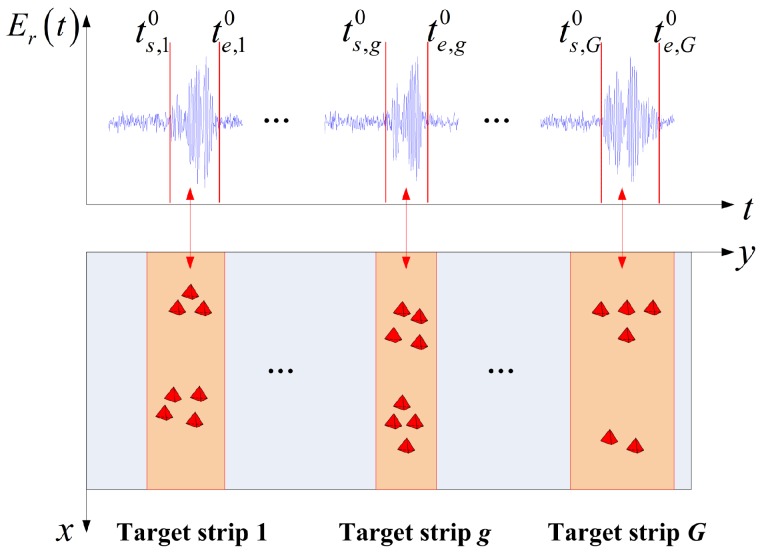
Received signal of the clustered targets in a narrow pulse MSCI. *G* is the number of target strips.

Discrete clustered targets are distributed in different range strips in the imaging region, and the target strip is defined as a strip with targets, as shown in Figure 2. The spacing between two target strips is larger than δy . These target strips are illuminated in different time periods when the pulse width of the waveform is narrow; thus, the system separates the echoes of the targets in the time domain. Figure 2 shows only the received signal for q=0. $t_{s,g}^{0},t_{e,g}^{0}\;\left(g=1,2,\cdots ,G\right)$ are the endpoints of the echoes for the targets in different target strips. The endpoints are located using the method introduced in Section 3.1, and the estimates are $t_{s,g},t_{e,g}\;\left(g=1,2,\cdots ,G\right)$. We define

(9) $$E_{r,g}={\left[E_{r,g}^{0},E_{r,g}^{1},\cdots ,E_{r,g}^{Q-1}\right]}^{T},$$

where $E_{r,g}^{q}=\left[E_{r}\left(t_{s,g}+\frac{T_{p}}{2}+qT\right),\cdots ,E_{r}\left(t_{k}+qT\right),\cdots ,E_{r}\left(t_{e,g}-\frac{T_{P}}{2}+qT\right)\right]$ , and $t_{k}$ is the sample time that satisfies $t_{s,g}+\frac{T_{P}}{2}⩽t_{k}⩽t_{e,g}-\frac{T_{P}}{2}$. The noise component of $E_{r,g}$ is $n_{g}$. By combining the detected endpoints $t_{s,g},t_{e,g}\;\left(g=1,2,\cdots ,G\right)$ and the imaging geometry, the target strips can be searched in an adaptive manner, as shown in Figure 2. In the *g*-th target strip, the dictionary $E_{rad,g}$ that corresponds to $E_{r,g}$ can be calculated according to Equation (6).

In the same target strip, the echoes of the targets overlap in the time domain; thus, the CIP combining $E_{rad,g}$ and the corresponding received signal vector $E_{r,g}$ is used to locate the regions with targets and achieve the target recovery results.

### 3.1. Location of the Target Strips

In the received signal $E_{r}\left(t\right)$, the echo $E^{sca}\left(t\right)$ is highly contaminated by noise n(t) at low signal-to-noise ratios (SNRs). However, the modified method based on spectral entropy proposed in this paper can locate the endpoints of the echoes to be free of non-echo regions using the difference between the spectrum of the echoes and that of noise.

The received signal for q=0 is divided into a sequence of frames with frame length $N_{0}$, and the signals in frame *m* are $s_{m0},s_{m1},\cdots ,s_{m(N_{0}-1)}$ . These signals are converted from the time domain to the frequency domain via fast Fourier transform (FFT), and $N_{FFT}$ is the total number of frequency components in the FFT; thus, $S_{m}\left(f_{0}\right),S_{m}\left(f_{1}\right),\cdots ,S_{m}\left(f_{\left(N_{FFT}-1\right)}\right)$ are obtained. We define

(10) $$es_{m}\left(f_{k}\right)={\left|S_{m}\left(f_{k}\right)\right|}^{\gamma }.$$

To improve the discriminability of the spectrum between echo and non-echo signals, γ should be greater than 1. Then, the probability distribution function (pdf) for the spectrum is estimated by normalizing the frequency components:

(11) $$P_{mi}=es_{m}\left(f_{i}\right)/\sum_{k=0}^{N_{FFT}-1}es_{m}\left(f_{k}\right),i=0,\ldots ,N_{FFT}-1.$$

The negative entropy [12] $H_{m}$ of frame *m* is defined as follows:

(12) $$H_{m}=\sum_{i=0}^{N_{0}-1}P_{mi}\log P_{mi}.$$

Here, $H_{m}$ is adjusted by subtracting $C_{H}$, the average negative entropy of the first 80 frames, and then the entropy features (EFs) to locate the endpoints is obtained as follows:

(13) $$EF_{m}={\left(H_{m}-C_{H}\right)}^{2}.$$

We define the contrast of EF as

(14) $$EF\_C=EF_{\max}/E,F_{mean},$$

where $EF_{\max}$ is the max EF, and $EF_{mean}$ is the average EF of the first 80 frames. A larger EF_C is favorable for finding the endpoints; thus, in the practical imaging process, we set

(15) $$\gamma =\arg\max_{\gamma }EF\_C\left(\gamma \right).$$

The estimated endpoints $t_{s,g},t_{e,g}\;\left(g=1,2,\cdots ,G\right)$ can be obtained using the algorithm presented in Section 3 of Reference [16]. Then, with the endpoints and configuration parameters of the imaging system, the target strips can be adaptively obtained. The location of the target strips is further analyzed in Section 4.1.

### 3.2. Location of the Regions with Targets in a Target Strip

According to [2], the imaging performance of MSCI depends on the self-correlation of $E_{rad}\left(\vec{r},t\right)$, i.e.,

(16) $$R_{I}\left(\vec{r},{\vec{r}}^{\prime }\right)=\frac{\left|\int E_{rad}\left(\vec{r},t\right)E_{rad}^{*}\left({\vec{r}}^{\prime },t\right)dt\right|}{\sqrt{\int E_{rad}\left(\vec{r},t\right)E_{rad}^{*}\left(\vec{r},t\right)dt}\sqrt{\int E_{rad}\left({\vec{r}}^{\prime },t\right)E_{rad}^{*}\left({\vec{r}}^{\prime },t\right)dt}}.$$

**Figure 3:**
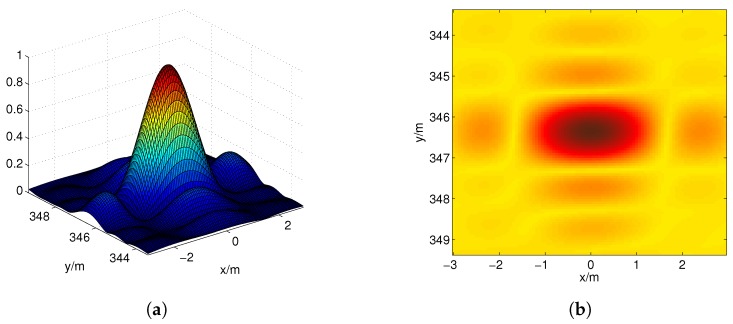
Coherence pattern. (**a**) three-dimensional view; and (**b**) plane view.

Figure 3 shows the coherence pattern $R_{I}\left(\vec{r},{\vec{r}}_{0}\right),$ where ${\vec{r}}_{0}$ is the center of the imaging region. The full width at half maximum in the *x*- and *y*-directions of the coherence pattern are $\Delta x_{0}$ and $\Delta y_{0}$, respectively. The scatterers in the main lobe of the coherence pattern cannot be easily resolved because of the high self-correlation of $E_{rad}\left(\vec{r},t\right)$ . Here, we select $\Delta x_{0}$ as the initial grid spacing.

In the case of a coarse grid spacing $\Delta x=\Delta y=\Delta x_{0}$, the *g*-th target strip is discretized as

(17) $$I_{L,g}^{\left(0\right)}=\left[{\vec{r}}_{g,1}^{\left(0\right)},{\vec{r}}_{g,2}^{\left(0\right)},\ldots ,{\vec{r}}_{g,l}^{\left(0\right)},\ldots ,{\vec{r}}_{g,L_{g,0}}^{\left(0\right)}\right],\;l=1,2,\ldots ,L_{g,0}.$$

Then, we can calculate $E_{rad,g}^{\left(0\right)}$, the dictionary corresponding to $E_{r,g}$ for the *g*-th (g=1,2,⋯,G) target strip, according to Equation (6). In this case, the gridding error $d^{\left(0\right)}=E_{r,g}-E_{rad,g}^{\left(0\right)}\sigma_{g}^{\left(0\right)}-n_{g}$ is large, so the MF reconstruction is introduced. The MF reconstruction algorithm has a low resolution but can adapt to the low-SNR and the large-gridding-error environments [13,17] to accurately locate the regions with targets. The scatter coefficients are estimated using the MF reconstruction algorithm as follows:

(18) $${\hat{\sigma }}_{g}^{\left(0\right)}={\left(E_{rad,g}^{\left(0\right)}\right)}^{H}E_{r,g},$$

where ${\left(E_{rad,g}^{\left(0\right)}\right)}^{H}$ is the conjugate transpose of the measurement matrix $E_{rad,g}^{\left(0\right)}$.

We set the threshold value ζ. If ${\hat{\sigma }}_{g,l}^{\left(0\right)}>\zeta {\hat{\sigma }}_{g,\max}^{\left(0\right)}$, there may be targets near the position ${\vec{r}}_{g,l}^{\left(0\right)}$, and denser grids will be built around it. To reduce the gridding error, we consider the fractional grid ZZFF=jjFF:j∈Z with a large integer F∈N, which is the refinement factor [14].

### 3.3. Target Reconstruction

#### 3.3.1. Grid Refinement

**Figure 4:**
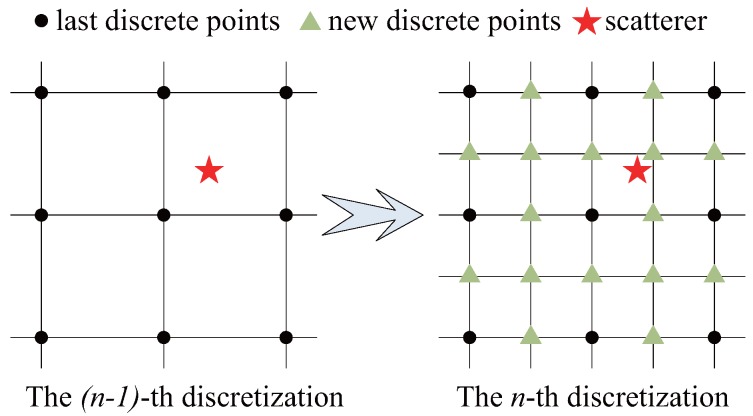
Grid refinement method.

We reduce the grid spacing in the regions with targets, Δx=ΔxΔx22,Δy=ΔyΔy22; the grid refinement method is shown in Figure 4.

Suppose that the measurement matrix $E_{rad,g}^{\left(n-1\right)}$ has been obtained from the (*n*−1)-th discretization; then, the *n*-th discretization increases the number of grids by $L_{g,n}$ whose positions are ${\vec{r}}_{g,l}^{\left(n\right)},l=1,\ldots ,L_{g,n}$. Hence,

(19) $$E_{rad,g}^{\left(n\right)}=\left[\begin{array}{cc} E_{rad,g}^{\left(n-1\right)} & E_{d,g}^{\left(n\right)} \end{array}\right],$$

where $E_{d,g}^{\left(n\right)}=\left[\begin{array}{cccc} \beta_{1}^{\left(n\right)} & \cdots & \begin{array}{cc} \beta_{l}^{\left(n\right)} & \cdots \end{array} & \beta_{L_{g,n}}^{\left(n\right)} \end{array}\right]\;$ and $\beta_{l}^{\left(n\right)}={\left[{\left(\beta_{l}^{\left(n\right)}\right)}^{0},{\left(\beta_{l}^{\left(n\right)}\right)}^{1},\cdots ,{\left(\beta_{l}^{\left(n\right)}\right)}^{Q-1}\right]}^{T}$, ${\left(\beta_{l}^{\left(n\right)}\right)}^{q}\;=\;\left[\begin{array}{cccc} E_{rad}\left({\vec{r}}_{g,l}^{\left(n\right)},t_{s,g}+\frac{T_{p}}{2}+qT\right) & \cdots & \begin{array}{cc} E_{rad}\left({\vec{r}}_{g,l}^{\left(n\right)},t_{k}+qT\right) & \cdots \end{array} & E_{rad}\left({\vec{r}}_{g,l}^{\left(n\right)},t_{e,g}-\frac{T_{p}}{2}+qT\right) \end{array}\right]$.

#### 3.3.2. Target Reconstruction Method

The grid refinement generates more grids than the measurements, and the scatterers of targets are often sparsely distributed; thus, sparse recovery is used. The orthogonal matching pursuit (OMP) is a suitable choice for target reconstruction because of its low computational complexity and accurate results in practical applications [18,19]. However, the off-grid scatterer will spill non-zero values into all grid-cells; in this case of denser grids, the increasing coherence of dictionary makes it difficult to ensure the OMP reconstruction, which relies on a low correlation of the columns of the measurement matrix.

Here, we introduce BE [14]. The discrete form of Equation (16) can be denoted by the following pairwise coherence:

(20) $$\mu \left(m,l\right)=\frac{\left|\langle \varphi_{m},\varphi_{l}\rangle \right|}{∥\varphi_{m}∥∥\varphi_{l}∥},$$

where $\varphi_{m}$ and $\varphi_{l}$ are the *m*-th and *l*-th column of $E_{rad,g}^{\left(n\right)}$, respectively. Let η>0 and define the η-coherence band of index *l* to be the set

(21) $$B_{\eta }\left(l\right)=\left\{m|\mu \left(m,l\right)>\eta \right\}$$

and the η-coherence band of the index set *S* is the set $B_{\eta }\left(S\right)=\cup_{l\in S}B_{\eta }\left(l\right)$. The double η-coherence band is defined as follows:

(22) $$B_{\eta }^{\left(2\right)}\left(S\right)\equiv B_{\eta }\left(B_{\eta }\left(S\right)\right).$$

When the scatterers in a cluster are sufficiently separated with respect to the size of the region represented by the double η-coherence band, the imaging performance will be significantly improved by inbedding BE into the OMP. Considering the double coherence band generated by grid refinement, the band-excluded orthogonal matching pursuit (BOMP) is stated in Algorithm 1. If η=1 is satisfactory, the BOMP is converted to the OMP; thus, the OMP is a special form of the BOMP, and the BOMP can adapt to a wider range of scenarios.

**Algorithm 1:** BOMP Algorithm.
**Input:** $E_{rad,g}^{\left(n\right)},E_{r,g},\eta >0$.**Initialization:** $x^{0}=0$, $r^{0}=E_{r,g}$, and $P^{0}=\emptyset$.**Iteration:** For j=1,…,s    **Step 1:** $i_{\max}=\arg\max_{i}\left|\langle r^{j-1},\varphi_{i}\rangle \right|,\;i\notin B_{\eta }^{\left(2\right)}\left(P^{j-1}\right)$;    **Step 2:** $P^{j}=P^{j-1}\cup \left\{i_{\max}\right\}$;    **Step 3:** $x^{j}=\arg\min_{z}{∥E_{rad,g}^{\left(n\right)}z-E_{r,g}∥}_{2}$ subject to $\mathrm{supp}\left(z\right)\in P^{j}$;    **Step 4:** $r^{j}=E_{r,g}-E_{rad,g}^{\left(n\right)}x^{j}$.**Output:** $x^{s}$.


To achieve the desired resolution, set n=n+1 and repeat from the grid refinement until ${∥E_{rad,g}^{\left(n\right)}x^{s}-E_{r,g}∥}_{2}⩽\varepsilon$ or $n=N_{\max}$; then, the reconstructed image is obtained for the *g*-th target strip, where g=1,2,⋯,G.

**Figure 5:**
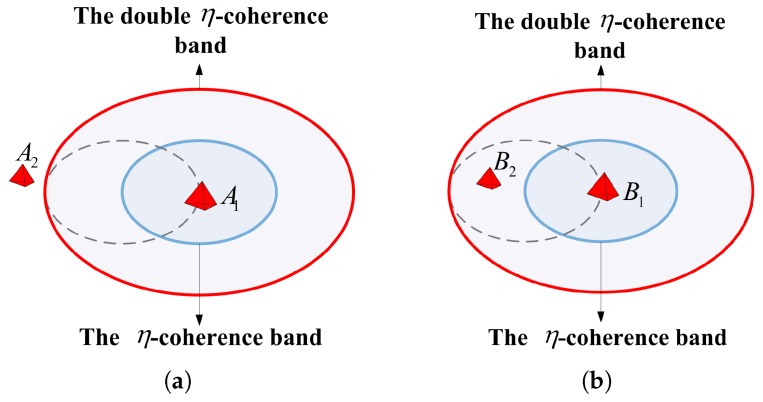
Coherence band and the scatterers. (**a**) the scatterer $A_{2}$ is outside the double η-coherence band of $A_{1}$; (**b**) the scatterer $B_{2}$ is in the double η-coherence band of $B_{1}$.

**Remark** **1.** The BOMP outperforms OMP in terms of maintaining the target profile in the recovery image. The condition for using the BOMP is that the scatterers in a cluster are sufficiently separated with respect to the size of the region represented by the double η-coherence band, as shown in Figure 5a. In Figure 5b, the scatterer $B_{2}$ is in the double η-coherence band of the scatterer $B_{1}$, so the scatterer $B_{2}$ will be excluded when performing Step 1 of the BOMP algorithm, which results in target reconstruction failure.

**Remark** **2.** For the n-th discretization, the computational cost of the OMP algorithm is approximately $O\left(s{K^{\prime }}_{g}{L^{\prime }}_{g,n}\right)$ [19], where $K_{g}^{\prime }$, $L_{g,n}^{\prime }$ are the numbers of the rows and columns of $E_{rad,g}^{\left(n\right)}$, respectively. The cost of BOMP increases to $O\left(s\left(N_{\eta b}+2\right){K^{\prime }}_{g}{L^{\prime }}_{g,n}\right)$ due to the introduction of BE, where $N_{\eta b}$ is the average of the columns in the η-coherence band of the object support. After another grid subdivision, $L_{g,n+1}^{\prime }=4L_{g,n}^{\prime }$. $N_{\eta b}$ also becomes four times the original value, so the total cost increases to approximately 16-fold.

## 4. Simulation Results and Discussion

Several simulation results are presented in this section to evaluate the effectiveness of the proposed approach. An X-band MSCI radar system with a carrier frequency of $f_{c}=9.3\;$ GHz is considered. The transceiver configuration is shown in Figure 1. The size of *D* is 4×1.5 m, and 36 transmitters are uniformly distributed in *D* with an inter-element spacing of d1=d2=0.5 m. The vertical distance *H* and angle θ are 600 m, $\pi /6$ rad, respectively.

The transmitters emit independent frequency-hopping waveforms with a bandwidth of 500 MHz:

(23) $$St_{i}\left(t\right)=\sum_{q=0}^{Q-1}rect\left(\frac{t-qT}{T_{p}}\right)\cdot \exp\left[j2\pi \left(f_{c}+f_{iq}\right)\left(t-qT\right)+\phi_{iq}\right],$$

where $T_{p}=20$ ns and T=10μs; Q=200 and $f_{iq},\phi_{iq}$ are the baseband frequency and initial phase of the *q*-th pulse of the *i*-th transmitter, respectively. The baseband frequency stochastically hops between 300 MHz and 800 MHz with *q*, and the initial phase is randomly set in the range of 0 to 2π . The received signal is mixed with zero-mean Gaussian noise, and the sampling frequency is 4 GHz. We set $\varepsilon =10^{-3}$, $N_{\max}=4$ and η=0.8 .

### 4.1. Location of the Target Strips

**Figure 6:**
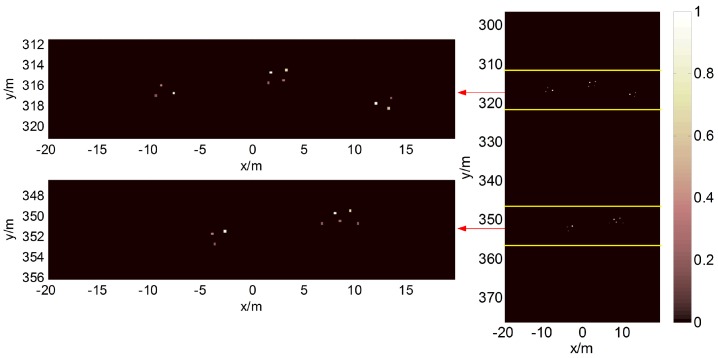
Target model in the simulation. The dynamic range is 6.

**Figure 7:**
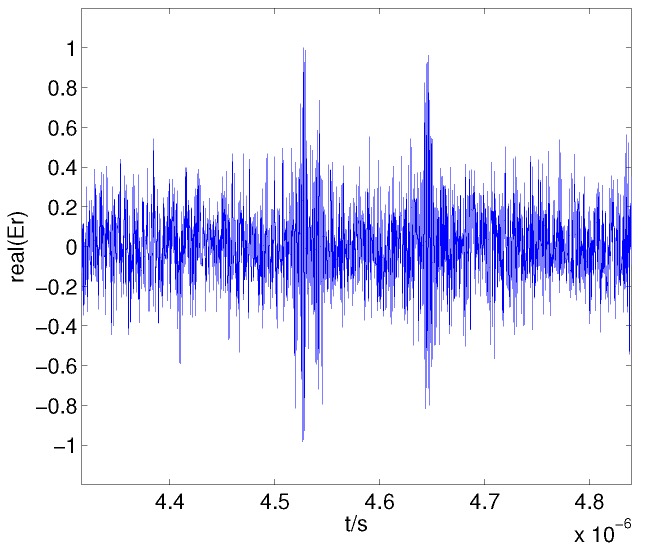
Received signal for a signal-to-noise ratio of 5 dB.

Using the target model in Figure 6, the received signal for an SNR of 5 dB can be obtained as shown in Figure 7; the echoes are highly contaminated by noise at low SNRs. However, the endpoints of the echoes can be located to be free of non-echo regions using the difference in their frequency spectra.

**Figure 8:**
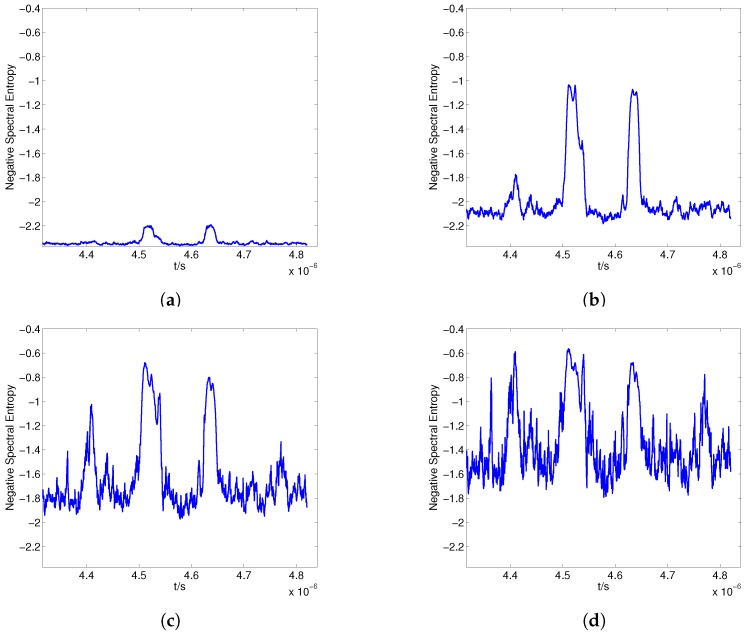
Negative spectral entropy for γ=1 (**a**); γ=3 (**b**); γ=5 (**c**); γ=7 (**d**).

The received signal is divided into a sequence of frames with frame length $N_{0}=80$, and the total number of frequency components in the FFT is $N_{FFT}=256$. The negative spectral entropy for different γ is shown in Figure 8. The spectrum amplitude of the non-echo segments is flat and lower than that of the echo segments; thus, the difference in $es_{m}\left(f\right)={\left|S_{m}\left(f\right)\right|}^{\gamma }$ between echo and non-echo segments increases when γ is larger than 1, which improves the discriminability of the negative entropy, as shown in Figure 8a,b. However, when γ increases further, the value of the negative spectral entropy in the non-echo regions fluctuates considerably, which is intractable for the endpoint detection, as shown in Figure 8c,d.

**Figure 9:**
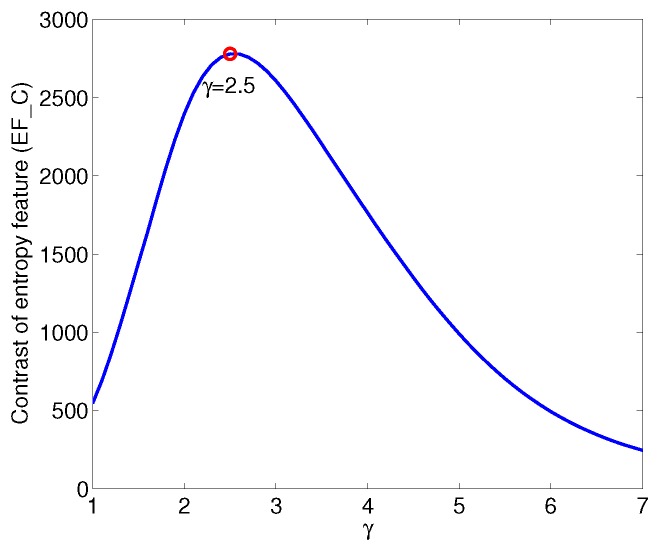
Contrast of entropy feature versus γ.

This paper locates the endpoints of the echoes according to the feature EF, and EF_C is used to measure the discriminability of EF between echo and non-echo segments. The performance curve of EF_C with respect to γ is obtained by increasing γ from 1 to 7, as shown in Figure 9. When 1⩽γ<2.5, the difference in EF between echo and non-echo segments increases with increases in γ, and the peak value is obtained when γ=2.5. However, when γ increases further, the negative spectral entropy of the non-echo segment fluctuates considerably, which causes a decrease in EF_C, consistent with the phenomenon shown in Figure 8.

**Figure 10:**
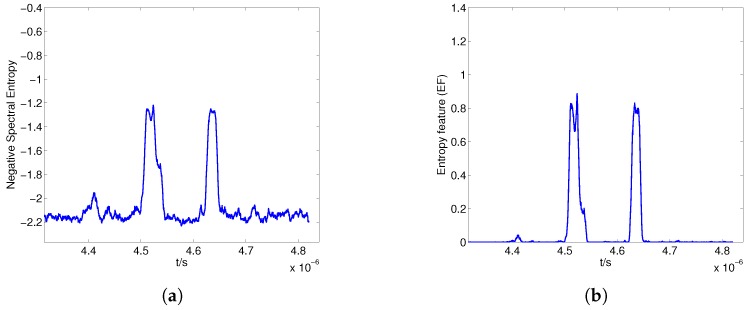
Negative spectral entropy (**a**) and entropy feature (**b**) for γ=2.5.

Figure 10 shows the negative spectral entropy and EF when γ=2.5. The negative spectral entropy of the non-echo segment is stable, and there is a clear difference in EF between the echo and non-echo segments, which is favorable for detecting the boundaries of the echo segments.

**Figure 11:**
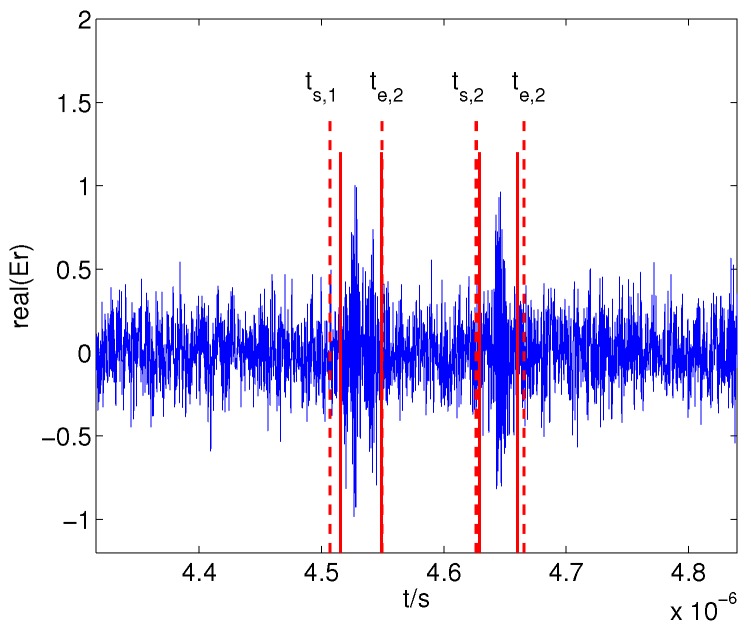
Detected boundaries, where $t_{s,1}=4.5070\times 10^{-6}$ s, $t_{e,1}=4.5495\times 10^{-6}$ s, $t_{s,2}=4.6265\times 10^{-6}$ s and $t_{e,2}=4.6658\times 10^{-6}$ s. The solid and dashed lines denote the true boundaries and detected boundaries, respectively.

Then, the detected beginning and ending boundaries of the echo segments can be obtained using the algorithm in Section 3 in [16], which are shown in Figure 11 and indicate the highly successful endpoint detection.

**Figure 12:**
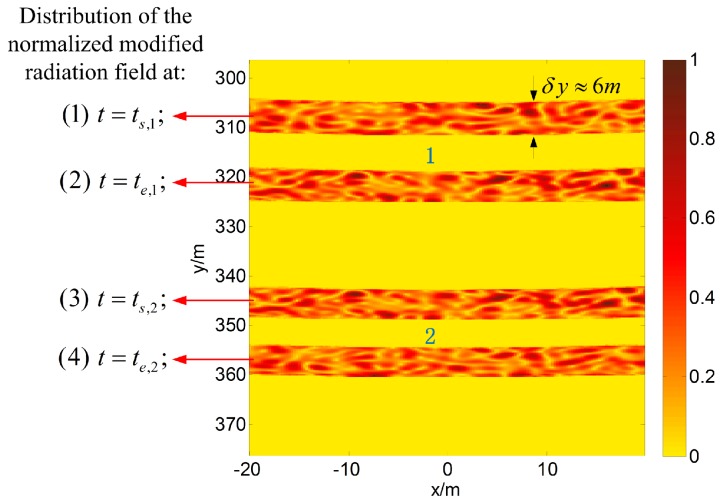
Distribution of the normalized modified radiation field and estimated target strips.

The distribution of the normalized modified radiation field at a given time is calculated according to the geometry of the imaging system. Figure 12 shows the normalized modified radiation field in the imaging region at $t=t_{s,1}$, $t=t_{e,1}$, $t=t_{s,2}$ and $t=t_{e,2}$. The modified radiation field begins to contact certain clustered targets when $t=t_{s,1}$, and it leaves the targets when $t=t_{e,1}$; thus, we can determine that Strip 1 may cover certain targets. In other words, Strip 1 is a target strip. Similarly, we know that Strip 2 is also a target strip.

### 4.2. Target Reconstruction

**Figure 13:**
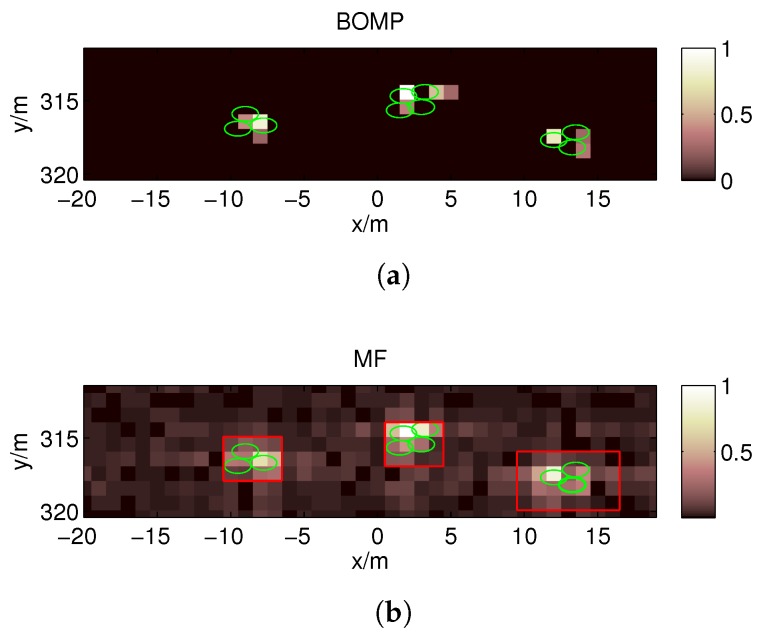
Imaging results using the band-excluded orthogonal matching pursuit algorithm (**a**) and the matched filter reconstruction algorithm (**b**) when *F* = 1 for the targets in the first target strip. The centers of the green ellipses denote the true positions of the scatterers.

**Figure 14:**
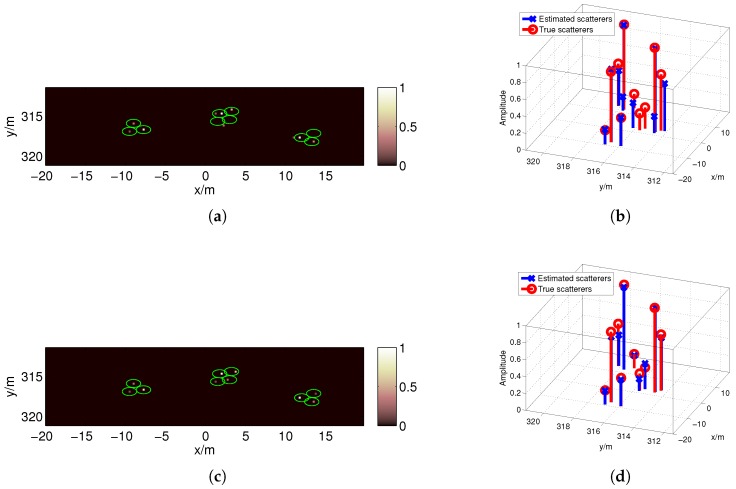
Imaging results by the OMP (**a**,**b**) and BOMP (**c**,**d**) when *F* = 4 for the targets in the first target strip. The centers of the green ellipses denote the true positions of the scatterers, and the regions represented by the double 0.8-coherence bands of the object supports are covered by the ellipses. The semi major axis and semi minor axis of each ellipse are 0.8 m and 0.5 m, respectively.

For the first target strip, the imaging result obtained using the BOMP algorithm in the coarse grids ($\Delta x_{0}=1$ m) is given in Figure 13a, which fails because the gridding error is as large as the echo data. However, in Figure 13b, the MF reconstruction algorithm is robust against gridding error; thus, the target regions can be estimated in the red boxes. Because ζ should be less than the reciprocal of the dynamic range $\sigma_{\max}/\sigma_{\min}=6$ , we set ζ=0.15 when estimating the target regions in this paper. Then, a denser grid is built around the estimated locations with a finer resolution. Figure 14a,b show the poor imaging result obtained by the OMP because, in practice, the scatterers are not located precisely on the pre-discretized grids, and the mutual coherence increases with *F* when the nearby columns of the measurement matrix become highly correlated. As expected, better recovery results are obtained by the BOMP algorithm in Figure 14c,d, where the object locations are accurately estimated, and the estimated scattering coefficients are more accurate than the OMP result.

**Figure 15:**
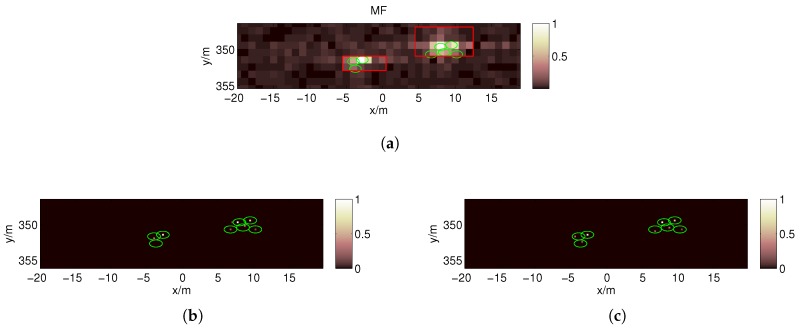
Imaging results of the MF reconstruction algorithm when *F* = 1 (**a**) and those of OMP (**b**) and BOMP (**c**) when *F* = 4 for the targets in the second target strip.

In the image reconstructed by the OMP in Figure 14a, false scatterers appear near the reconstructed strong scatterers. Due to the introduction of BE, the false scatterers are eliminated, and a more accurate reconstructed image can be obtained using the BOMP, as shown in Figure 14c. Similarly, in the second target strip, the target regions can be estimated in the red boxes shown in Figure 15a, and the BOMP yields a better recovery result than the OMP for the targets, as shown in Figure 15b,c.

For the target model in Figure 6, we can determine that the targets are distributed in the red boxes in Figure 13b and Figure 15a after locating the target strips and target reconstruction by the MF in the coarse grids. Then, only the regions in the red boxes, which represent just a tiny fraction of the imaging region, are discretized to a fine grid. Thus, the proposed method can significantly reduce the column number of the measurement matrix in Equation (6) and is more computationally efficient than the method using a uniform fine grid for the entire imaging region.

### 4.3. Performance Simulations

We randomly set 100 discrete clustered target models with a dynamic range of 6, and the spacing of the scatterers in a cluster is greater than the size of the green ellipses in Figure 14a,c. In the target models, all scatterers are distributed in Strips 1 and 2, as shown in Figure 12.

An endpoint detection is counted as a success if $0⩽t_{s,g}^{0}-t_{s,g}⩽\frac{T_{p}}{2}$ and $0⩽t_{s,g}^{0}-t_{s,g}⩽\frac{T_{p}}{2}\;\left(g=1,2\right)$. For the 100 target models, the success rates of endpoint detection with respect to different levels of white noise are shown in Table 1. The success rates when $\gamma =\arg\max_{\gamma }EF\_C\left(\gamma \right)$ are higher than those when γ is fixed at 1 or 5.

**Figure 16:**
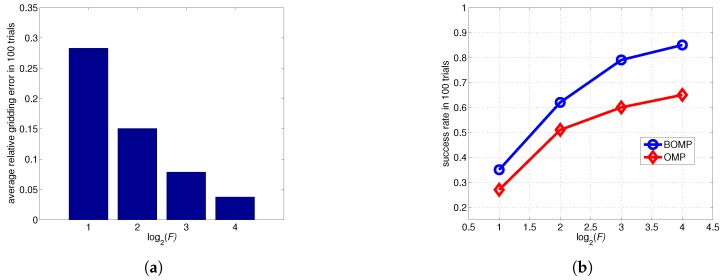
Results of the imaging performance experiments. (**a**) the relative gridding error is approximately inversely proportional to the refinement factor; and (**b**) success probability for target reconstruction by the BOMP and OMP versus the refinement factor *F*. A reconstruction is considered successful if every reconstructed object is in the double 0.8-coherence band of the object support. The SNR is fixed at 5 dB.

To further evaluate the imaging performance, we perform grid subdivision four times. The refinement factor *F* increases from 2 to 16, and the performance curve of the probability of success with respect to *F* is obtained, as shown in Figure 16b. It can be seen that success rate rises with *F*, and the BOMP outperforms the OMP because of the introduction of BE. Figure 16a shows that the relative gridding error ${∥d∥}_{2}/{∥E_{r}∥}_{2}$ is approximately inversely proportional to the refinement factor.

## 5. Conclusions

MSCI is based on the temporal-spatial stochastic radiation field and can achieve ultra-high resolution in real aperture staring radar imaging under all-weather and all-day circumstances. This paper proposes an adaptive MSCI for targets that appear in discrete clusters. The adaptive MSCI can locate the target strips in an adaptive manner and adaptively estimate the regions with targets in a target strip; thus, a uniform fine grid in the entire imaging area is avoided, and the complexity of the imaging process is reduced. Then, the applicability of MSCI is significantly improved. With several numerical simulations, the proposed approach is proven to be effective and its performance is analyzed. The adaptive imaging method for lower SNR conditions is worthy of further consideration.

## Figures and Tables

**Table 1:** Success rates of endpoint detection for different noise levels.

γ	Success Rate (%)		
	0 dB	3 dB	5 dB
1	75	96	98
5	92	95	99
$\gamma =\arg\max_{\gamma }EF\_C\left(\gamma \right)$	99	100	100
